# An overview of the multi-dimensional mechanisms of exercise-regulated hormones and growth factors in cardiac physiological adaptation

**DOI:** 10.3389/fphys.2025.1642389

**Published:** 2025-09-10

**Authors:** Shuaiwang Huang, Zhanglin Chen, Haoming Li, Lan Zheng, Zuoqiong Zhou, Xiyang Peng, Changfa Tang

**Affiliations:** Key Laboratory of Physical Fitness and Exercise Rehabilitation of Hunan Province, College of Physical Education, Hunan Normal University, Changsha, China

**Keywords:** physiological cardiac hypertrophy, exercise, hormones, growth factors, mitochondria

## Abstract

Physiological cardiac hypertrophy represents an adaptive response of the heart to chronic physiological stimuli, including sustained exercise, and is characterized by cardiomyocyte enlargement and structural optimization to enhance pumping efficiency. While several studies on cardiac physiological adaptation have been published recently, a systematic integration of information on exercise-regulated hormonal and growth factor networks remains lacking. To address this limitation, toward the systematization of a ‘multi-dimensional mechanism’ model, here we review the molecular mechanisms underlying exercise-induced physiological cardiac hypertrophy, with particular focus on how physical activity regulates hormones and growth factors including insulin-like growth factor-1, vascular endothelial growth factor, neuregulin-1, and norepinephrine. These mediators activate intricate signaling pathway networks that promote protein synthesis in cardiomyocytes, strengthen myocardial contractility, and induce angiogenesis. The highlighted findings not only provide novel insights into the cardioprotective mechanisms of exercise but also identify potential biomarkers that enable the development of precision exercise prescriptions tailored to individuals with cardiovascular diseases.

## 1 Introduction

Cardiovascular diseases (CVDs) remain the leading cause of global mortality (Naghavi et al., 2024). Approximately 20.5 million deaths were reported in 2021—accounting for one-third of global mortality—as was a substantial increase of over 6 million cases between 1990 and 2019, as per the 2023 World Heart Federation (Naghavi et al., 2024). Physical inactivity is a key modifiable risk factor contributing to the global burden of CVD. Physiological cardiac hypertrophy is defined as an adaptive myocardial adaptation(physiological) process driven by hemodynamic demands during physiological challenges such as chronic exercise and pregnancy (Weeks and McMullen, 2011). This non-pathological adaptation involves cardiomyocyte enlargement with concomitant increased myofibril density and diameter, resulting in enhanced contractile function and cardiac output. Key features include preserved or mildly elevated ejection fraction and coronary reserve, proportional angiogenesis, increased myoglobin expression, and the absence of pathological markers such as myocardial fibrosis or necrosis (Qiu et al., 2022). Furthermore, it is accompanied by increased mitochondrial biogenesis and enhanced mitochondrial function (Abel and Doenst, 2011). Importantly, this hypertrophic response, with cardioprotective benefits, is reversible (Qiu et al., 2022). Given the critical role of physiological cardiac hypertrophy in cardiovascular adaptation, elucidating its regulatory mechanisms is a research priority.

Exercise, as a non-invasive intervention, is globally recommended for both preventing and managing CVD. In addition to improving myocardial contractility and endurance capacity, chronic exercise promotes structural cardiac adaptation, with physiological hypertrophy serving as its hallmark adaptation. Dynamic fluctuations in circulating hormones and cytokines during acute exercise and recovery phases act as key mediators of exercise-induced cardiac adaptation (Qiu et al., 2022). These bioactive molecules activate signaling pathways that regulate cardiomyocyte proliferation, differentiation, and metabolic adaptation, thereby inducing beneficial hypertrophy (Waring et al., 2014; Chen et al., 2021). Notably, hormonal and growth factor responses exhibit marked sensitivity to exercise type, intensity, and duration, suggesting the existence of a sophisticated molecular regulatory network. Therefore, studying the exercise-mediated regulation of such biomolecules offers dual benefits: advancing our understanding of cardiac adaptation mechanisms and guiding the design of personalized exercise regimens for cardiovascular rehabilitation. Although existing research has predominantly focused on isolated hormonal pathways, critical knowledge gaps persist regarding (1) the dynamic synergistic regulation of exercise-induced hormonal networks; (2) the dose-response relationships of specific exercise modalities in targeted populations (e.g., individuals with diabetes); and (3) the mechanistic interplay between lymphangiogenesis and fibrotic thresholds. In this study, we address these unresolved questions through multi-dimensional mechanistic integration. This encompasses the hierarchical integration of molecular, cellular, and systemic adaptations orchestrated by exercise-regulated hormonal networks, spanning the following four interconnected dimensions: (1) the molecular dimension involving cross-talk between key signaling pathways activated by hormones and growth factors; (2) the cellular dimension coordinating responses across cardiomyocytes, endothelial cells, and fibroblasts; (3) the temporal dimension reflecting dynamic hormone fluctuations during acute exercise versus chronic training; and (4) the systemic dimension integrating endocrine, exercise, and hemodynamic stimuli. These dimensions function synergistically rather than additively, forming an adaptive network that scales with exercise intensity and duration.

## 2 Exercise-induced myocardial proliferation and growth

**FIGURE 1:**
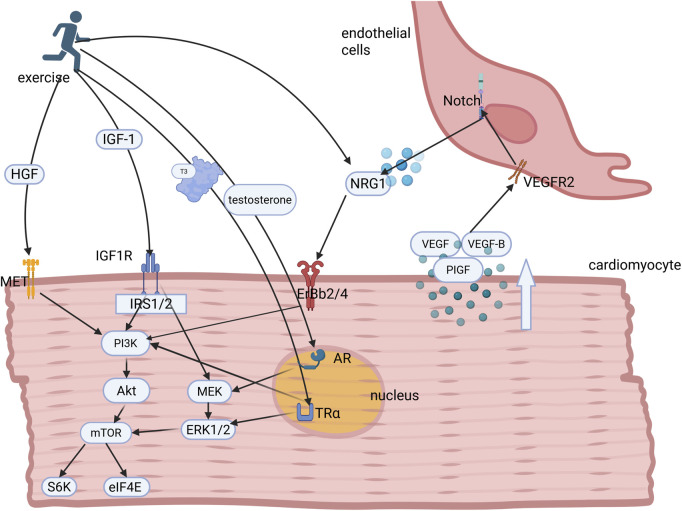
Hormonal and growth factor regulation underlying exercise-induced myocardial proliferation and growth. Exercise stimulates the secretion of key hormones and growth factors, including IGF-1, HGF, testosterone, NRG1, and triiodothyronine (T3), which bind to their cognate receptors to activate downstream PI3K/AKT and MEK/ERK1/2 signaling cascades. These pathways collectively orchestrate cardiomyocyte proliferation and hypertrophic growth. Concomitantly, exercise induces paracrine VEGF secretion from cardiomyocytes, which binds to endothelial cell surface receptors to initiate Notch signaling. This intercellular crosstalk promotes endothelial NRG1 release, which together with circulatory NRG1, amplifies ErbB-mediated signaling in cardiomyocytes, thereby establishing a coordinated microenvironment for adaptive cardiac adaptation. IGF-1, insulin-like growth factor-1; IRS1, insulin receptor substrates 1; PI3K, phosphoinositide 3-kinase; AKT, protein kinase B; ERKs, extracellular signal-regulated kinases; MEK, mitogen-activated protein kinase; NRG1, neuregulin-1; HGF, hepatocyte growth factor; VEGF, vascular endothelial growth factor.

Prolonged exercise training leads to morphologic adaptations typical of the athlete’s heart syndrome, including the progressive volumetric expansion of cardiomyocytes, with increased sarcomeric diameter (Hastings et al., 2024). This adaptation process primarily manifests as left ventricular hypertrophy proportional to the exercise intensity and the duration of cumulative training, within established physiological limits (Kemi et al., 2005). This adaptive transformation involves the coordinated activation of endocrine and paracrine signaling pathways (Figure 1). Specifically, hormonal mediators and growth factors cooperatively regulate the molecular mechanisms that enhance myocardial contractile performance, improve metabolic substrate utilization, and increase cardiac functional reserve. These integrative adaptations collectively enable the cardiovascular system to meet elevated metabolic demands during sustained physical activity while maintaining the hemodynamic equilibrium.

### 2.1 Insulin-like growth Factor-1 (IGF-1)

IGF-1, a multifunctional peptide hormone, regulates cardiac metabolic homeostasis, hypertrophic adaptation, cellular senescence, and apoptosis through IGF-1 receptor (IGF1R)-mediated signaling pathways (Troncoso et al., 2014). Previous studies have reported that moderate-intensity aerobic exercise enhances cardiac expression of IGF-1 and IGF1R (Weeks et al., 2017; Cheng et al., 2013). For instance, a 4-week swimming training protocol significantly increased myocardial IGF-1 mRNA levels in zebrafish (Chen et al., 2021). have, murine models with partial IGF-1 deficiency exhibit impaired cardiac function and fibrotic remodeling(pathological) (González-Guerra et al., 2017). Mechanistically, IGF-1 regulates cardiac mass and function via insulin receptor substrates 1 (IRS1) and 2, as genetic knockout of both IRS isoforms abolishes exercise-induced physiological hypertrophy (Riehle et al., 2014). The phosphoinositide 3-kinase (PI3K)/protein kinase B (Akt) axis, a key downstream target of IRS signaling, controls myocardial growth dynamics. Dysregulation of this pathway, characterized by reduced PI3K activation and increased Akt dephosphorylation, significantly compromises cardiac adaptation to exercise (Riehle et al., 2014).

Furthermore, extracellular signal-regulated kinases (ERKs) serve as complementary signaling mediators for IGF-1-induced physiological cardiomyocyte hypertrophy. These mitogen-activated protein kinases play dual roles in physiological and pathological cardiac remodeling (Gallo et al., 2019). Notably, the mitogen-activated protein kinase (MEK)/ERK cascade interacts synergistically with PI3K/Akt signaling to coordinate the transcriptional regulation of cardiomyocyte growth and proliferation, forming an integrated signaling network that modulates hypertrophic responses to hemodynamic stress (Chattergoon et al., 2014; Sundgren et al., 2003).

### 2.2 Testosterone

Testosterone, a steroid hormone produced in Leydig cells, the ovaries, and the adrenal cortex, has cardioprotective effects by mitigating fibrotic remodeling and oxidative stress (Bianchi, 2018). Acute high-intensity resistance exercise induces rapid testosterone surges, primarily mediated by activating the hypothalamic-pituitary-gonadal axis, and transient decreases in plasma sex hormone-binding globulin levels during intense physical exertion (Vingren et al., 2010). Androgen signaling enhances cardiac IGF-1 expression, with testosterone supplementation inducing dose-dependent increases in myocardial mass and IGF-1 content in preclinical models (Zebrowska et al., 2017). At the molecular level, testosterone interacts with nuclear androgen receptors (AR) in cardiomyocytes (Jänne et al., 1993), triggering MEK/ERK1/2 signaling, which activates the mTORC1/S6K1 pathway, ultimately driving physiological hypertrophy development (Altamirano et al., 2009).

### 2.3 Thyroid hormones

Thyroid hormones, which are essential nuclear receptor ligands for cardiac morphogenesis and metabolic regulation, primarily act through the peripheral conversion of thyroxine (T4) to bioactive triiodothyronine (T3), mediated by type 2 deiodinase (Mullur et al., 2014). Acute moderate-to-vigorous aerobic exercise induces transient increases in serum T3, T4, and thyroid-stimulating hormone levels (Hackney and Saeidi, 2019). These hormones bind to cardiac thyroid hormone receptors TRα1 (localized in the nucleus and cytoplasm) and TRβ1 (Mullur et al., 2014). In turn, TRα1 initiates rapid PI3K activation and subsequent Akt-mTOR-S6K pathway stimulation following T3 binding (K et al., 2006). Simultaneously, T3 promotes ERK phosphorylation in cardiomyocytes, establishing a synergistic signaling mechanism that enhances protein synthesis and contractile machinery adaptation, ultimately improving cardiac contractility (Chattergoon et al., 2014).

### 2.4 Neuregulin-1(NRG1)

NRG1, a key member of the epidermal growth factor family in the cardiovascular system (Falls, 2003), modulates multiple cardiac processes, including myocardial metabolism, cellular proliferation, and regeneration. Chronic exercise training enhances NRG1/ErbB signaling (Cai et al., 2016), since pharmacological inhibition of this pathway abolishes exercise-mediated cardiac repair in rodent models (Cai et al., 2016). Specifically, endothelial-derived NRG1 acts via paracrine signaling, binding to ErbB3/ErbB4 receptors on neighboring cardiomyocytes to initiate ErbB2 heterodimer formation (Odiete et al., 2012). These receptor complexes, particularly ErbB2/ErbB4 heterodimers, are critical for cardiomyocyte proliferation by activating downstream PI3K/Akt signaling, which, in turn, coordinates ventricular myocyte differentiation and hypertrophic growth (Zhao et al., 1998).

### 2.5 Hepatocyte growth factor (HGF)

HGF has multifunctional cardioprotective effects, including th inhibiting apoptosis and autophagy, promoting angiogenesis, suppressing fibrosis and inflammation, regulating immune function, and stimulating cardiomyocyte regeneration (Arechederra et al., 2013; Gallo et al., 2015). Chronic aerobic exercise induces the significant upregulation of myocardial HGF expression (Zhang et al., 2021). Mechanistically, HGF signaling is mediated by c-Met tyrosine kinase receptors. Upon ligand binding, receptor autophosphorylation initiates activation of the PI3K/Akt signaling cascade (Gallo et al., 2015; Gallo et al., 2014). Notably, transgenic HGF overexpression improves post-myocardial infarction recovery in murine models by enhancing angiogenesis, reducing cardiomyocyte apoptosis, and restoring ventricular contractile function (Jayasankar et al., 2005).

Emerging evidence indicates that diverse exercise paradigms elicit distinct endocrine and cardiovascular adjustments across various demographic groups, potentially influencing myocardial proliferation and growth outcomes. Consequently, exercise prescriptions should be personalized according to individual health profiles. In patients recovering from myocardial infarction, low-intensity aerobic training predominantly elevates IGF-1 and NRG1 levels while mitigating exercise-induced cardiovascular risks (Cai et al., 2016; Tan et al., 2023). In normotensive individuals, both acute and chronic aerobic or resistance training foster cardiovascular adaptation, with high-intensity resistance training demonstrating superior efficacy in enhancing anabolic hormone profiles (IGF-1, testosterone) (Grubb et al., 2014; Seo et al., 2018), whereas HGF reaches peak levels following prolonged endurance exercise (Bonsignore et al., 1985). Notably, obese populations experience acute exercise-induced endocrine dysregulation, marked by heightened catecholamine responses and aberrant fluctuations in testosterone, growth hormone, and thyroxine (Hansen et al., 2012). In contrast, systematic exercise training restores endocrine homeostasis, significantly improving hormonal balance and metabolic regulation in this demographic (Hansen et al., 2012).

## 3 Exercise-induced cardiovasculogenesis and lymphangiogenesis

Physical training enhances coronary vasodilation, improves myocardial perfusion, and stimulates capillary network expansion through neovascularization and collateral vessel formation. Cardiac lymphatic vessels regulate interstitial fluid clearance, directing the subendocardial drainage toward the epicardial collectors, which ultimately drain via mediastinal lymph nodes into the venous system (Liu and Oliver, 2023). Exercise-induced lymphangiogenesis serves as an adaptive mechanism that alleviates inflammatory cell infiltration, suppresses fibrotic remodeling, and reduces myocardial edema (Henri et al., 2016). Furthermore, itprovides therapeutic benefits in ischemic cardiomyopathy (Shimizu et al., 2018).

**FIGURE 2:**
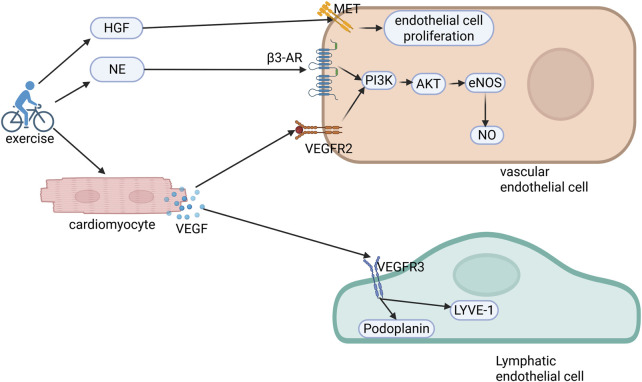
Molecular mechanisms of exercise-induced cardiovascular and lymphatic angiogenesis. Exercise elevates circulating hepatocyte growth factor (HGF) and norepinephrine (NE) levels while stimulating cardiomyocyte-derived vascular endothelial growth factor (VEGF) secretion. HGF binds to c-Met receptors on endothelial cells, facilitating their proliferation and migration. Concurrently, NE and VEGF engage β3-adrenergic receptors (β3-AR) and VEGFR2, respectively, activating the PI3K/AKT/eNOS/NO signaling axis to orchestrate coronary angiogenesis. Furthermore, VEGF interacts with VEGFR3 on lymphatic endothelial cells, upregulating the lymphangiogenic markers LYVE-1 and podoplanin, thereby establishing a dual regulatory network of coordinated vascular and lymphatic adaptation. PI3K, phosphoinositide 3-kinase; AKT, protein kinase B; eNOS, endothelial nitric oxide synthase; NO, nitric oxide.

This coordinated vascular-lymphatic adaptation is regulated by exercise-modulated catecholamines and growth factors such as vascular endothelial growth factor (VEGF) and HGF (Figure 2).

### 3.1 VEGF

The VEGF family coordinates vascular and lymphatic development through receptor-specific interactions: VEGF-A, VEGF-B, and placental growth factor (PlGF) bind VEGFR1, whereas VEGF-C and VEGF-D selectively activate VEGFR3 (Ferrara et al., 2003). VEGFR2 serves as the pivotal receptor orchestrating angiogenesis (Kappas et al., 2008). Under VEGFR1 deficiency or elevated VEGF-B/PlGF bioavailability, VEGF exhibits enhanced binding affinity to VEGFR2 leading to a higher activation efficacy and amplified angiogenic processes (Shibuya, 2006). Exercise induces cardiomyocyte-derived VEGF paracrine signaling, which activates endothelial VEGFRs to stimulate the PI3K/Akt and endothelial nitric oxide synthase (eNOS)/nitric oxide (NO) pathways (Wilson et al., 2015; Dimmeler et al., 1999). The eNOS/NO axis plays a pivotal role in coronary angiogenesis and cardioprotection (Bernardo et al., 2018), with Akt-dependent eNOS phosphorylation serving as a central regulatory mechanism (Dimmeler et al., 1999). Coronary angiogenesis modulates hypertrophic responses, as evidenced by endothelial VEGFR2/Notch-dependent NRG1 release, promoting physiological hypertrophy (Kivelä et al., 2019).

VEGF-C and VEGF-D are primary mediators of exercise-induced lymphangiogenesis. Recent studies found that LYVE-1 facilitates lymphatic endothelial cell (LEC) migration via integrin α9β1 signaling (Capuano et al., 2019), whereas podoplanin mediates lymphatic lumen formation through CLEC-2 receptor-dependent mechanisms (Suzuki-Inoue et al., 2018). In murine models, swimming and eccentric training upregulate cardiac VEGF-C and VEGF-D expression (Bei et al., 2022), which bind VEGFR3 onLECs to enhance lymphatic density and upregulate the lymphangiogenic markers LYVE-1 and podoplanin. Pharmacological VEGFR3 inhibition blocks exercise-mediated lymphangiogenesis (Bei et al., 2022), while LYVE-1 facilitates endothelial migration (Wu et al., 2014). Podoplanin, a LEC-specific glycoprotein, controls lymphatic morphogenesis, as its deficiency causes lymphatic maldevelopment and nodal edema (Schacht et al., 2003).

### 3.2 HGF

HGF has pleiotropic effects on cardiovascular homeostasis through its high-affinity receptor, c-Met tyrosine kinase. Mechanistically, HGF stimulates endothelial cell proliferation and migration while suppressing apoptosis via PI3K/Akt and MAPK/ERK signaling cascades, thereby promoting neovascularization (Bussolino et al., 1992). This pro-angiogenic action is amplified through synergistic interactions with VEGF and angiopoietin-1, which collectively stabilize nascent vessels by recruiting pericytes and enhancing endothelial barrier function (Gallo et al., 2015). In preclinical chronic ischemic models (e.g., porcine myocardial infarction), intramyocardial HGF administration increases capillary density by 30%–40% and improves regional blood flow, as quantified using microsphere perfusion assays. These benefits extend to functional outcomes, with HGF-treated animals exhibiting enhanced left ventricular ejection fraction and reduced infarct size (Yuan et al., 2012). However, HGF’s role is context-dependent: while beneficial in ischemia, HGF exacerbates tumor angiogenesis and atherosclerotic plaque vulnerability by upregulating matrix metalloproteinases (MMPs) and promoting intraplaque neovascularization (Abounader and Laterra, 2005; Ma et al., 2002).

### 3.3 Epinephrine and norepinephrine

Exercise-induced catecholamine release mediates sympatho-adrenal activation, enhancing cardiac output through chronotropic and inotropic effects while regulating vascular tone (Motiejunaite et al., 2021). Chronic activation of the endothelial β3-adrenergic receptor (β3-AR) represents a cardioprotective mechanism. β3-AR signaling stimulates eNOS through its phosphorylation at Ser1177 and dephosphorylation at Thr495, amplifying NO production without altering eNOS expression (Calvert et al., 2011). Notably, adrenaline-deficient mice develop pathological left ventricular hypertrophy after 6 weeks of treadmill training, characterized by interstitial fibrosis and impaired diastolic function; this phenotype is rescued by β3-AR agonist treatment (Mendes et al., 2018). These findings highlight β3-AR’s unique role in balancing exercise-induced hemodynamic stress and adaptive vascular growth.

Exercise triggers a complex molecular cascade that regulates coronary and lymphatic vascular development. The VEGF family (VEGF-A/B, PlGF, and VEGF-C/D) and their receptors (VEGFR1-3) coordinate angiogenesis and lymphangiogenesis (Ferrara et al., 2003). VEGFR2 is pivotal for angiogenesis, with heightened activity under VEGFR1 suppression or elevated VEGF-B/PlGF signaling (Kappas et al., 2008; Shibuya, 2006). Cardiomyocyte-derived VEGF activates endothelial VEGFRs, initiating PI3K/Akt and eNOS/NO pathways critical for coronary angiogenesis and cardioprotection (Wilson et al., 2015; Dimmeler et al., 1999). VEGF-C/D drive lymphangiogenesis via LYVE-1-mediated endothelial migration and podoplanin-dependent lumen formation (Bei et al., 2022). HGF complements these effects by promoting endothelial proliferation/migration and neovascularization through PI3K/Akt and MAPK/ERK pathways, synergizing with VEGF and angiopoietin-1 to enhance outcomes (beneficial in ischemia but risk-augmenting in tumors/atherosclerosis) (Gallo et al., 2015; Bussolino et al., 1992). Catecholamines (epinephrine/norepinephrine) released during exercise activate sympatho-adrenal signaling via β3-ARs, boosting cardiac output, modulating vascular tone, and amplifying NO production via eNOS activation, reinforcing cardioprotection (Calvert et al., 2011; Mendes et al., 2018). While exercise improves coronary perfusion, prolonged endurance training may induce maladaptive coronary changes (Lin et al., 2017). Optimizing exercise protocols for coronary disease requires precision medicine—tailoring regimens using dose-response modeling, biomarkers, and psychosocial profiling to maximize therapeutic benefits while mitigating risks.

## 4 Exercise-mediated mitochondrial adaptation and metabolic reprogramming

**FIGURE 3:**
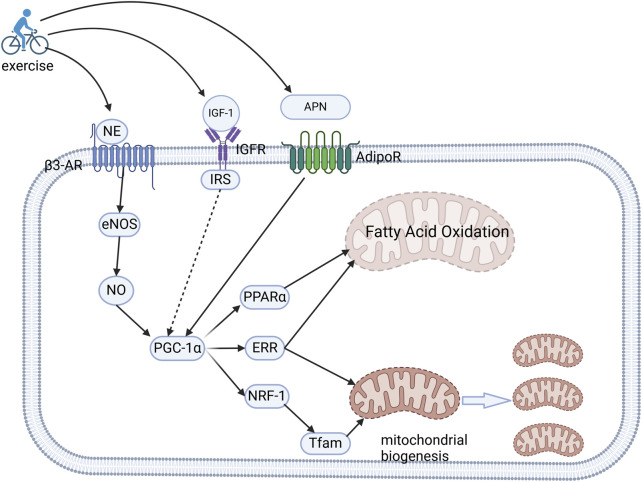
Molecular mechanisms of exercise-induced mitochondrial adaptation and metabolic optimization. Exercise upregulates the cardiac expression of NE, IGF-1, and APN. NE engagesβ3-AR to activate the eNOS/NO signaling axis, subsequently amplifying the expression of PGC-1α. Concurrently, IGF-1 and APN stimulate the activation of PGC-1α through their respective receptors. This transcriptional coactivator orchestrates mitochondrial reprogramming by synergizing with PPARα and ERRs to enhance fatty acid β-oxidation, while also collaborating with NRF-1 and ERRs to upregulate the expression of Tfam. This coordinated regulation drives mitochondrial DNA transcription and biogenesis, and consequently energy substrate optimization and enhanced oxidative capacity.NE, norepinephrine; IGF-1, insulin-like growth factor-1; APN, adiponectin; β3-AR, β3-adrenergic receptors; PGC-1α, peroxisome proliferator-activated receptor gamma coactivator-1α; ERRs, estrogen-related receptors; NRF-1, nuclear respiratory factor-1; Tfam, mitochondrial transcription factor A.

Mitochondrial dysfunction is a hallmark of CVD. Importantly, moderate exercise activates mitochondrial adaptation through enhanced respiratory chain activity, and improved quality control (biogenesis, mitophagy, and fusion/fission dynamics), leading to the maintenance of cellular homeostasis (Guan et al., 2019; Campos et al., 2017). For instance, a 3-week endurance training program was shown to normalize the redox balance and restore mitochondrial efficiency in a high-fat diet-induced rodent model (Tocantins et al., 2023). Central to this adaptation is the peroxisome proliferator-activated receptor gamma coactivator-1α (PGC-1α), a master transcriptional regulator abundantly expressed in cardiomyocytes. PGC-1α coordinates mitochondrial biogenesis by interacting with nuclear receptors (peroxisome proliferator-activated receptor α[PPARs] and estrogen-related receptor [ERRs]) and transcription factors (NRF-1/2), thereby promoting the oxidative phosphorylation capacity, fatty acid β-oxidation, and mitochondrial DNA replication (Figure 3) (Finck and Kelly, 2006; Lehman et al., 2000). Furthermore, the synergistic interaction between ERRα and PGC-1α fine-tunes mitochondrial gene networks to ensure metabolic flexibility (Schreiber et al., 2004).

### 4.1 Epinephrine and norepinephrine

Exercise-induced sympathoadrenal activation elevates circulating catecholamines (epinephrine and norepinephrine) and upregulates cardiacβ3-AR expression. β3-AR signaling enhances the activity of eNOS through two post-translational modifications: the phosphorylation of Ser1177 (activation) and the dephosphorylation of Thr495 (inactivation); these collectively amplify eNOS-derived NO production without altering total eNOS protein levels (Calvert et al., 2011). The resultant NO/cGMP signaling cascade activates PGC-1α, NRF-1, and mitochondrial transcription factor A, driving mitochondrial biogenesis and respiratory chain optimization (Nisoli et al., 2004). This pathway is indispensable for exercise-induced metabolic adaptation, as evidenced by eNOS-knockout mice, which fail to show mitochondrial proliferation or improved oxidative capacity following training (Nisoli et al., 2003; Vettor et al., 2014). The age-associated decline in mitochondrial integrity observed in cardiovascular pathologies may be associated with β3-AR downregulation.

### 4.2 IGF-1

IGF-1, elevated in response to both acute and chronic exercise, coordinates myocardial energy substrate utilization via IRS-mediated pathways. IRS1/2 are critical adapters linking IGF-1 receptor activation to downstream effectors. Notably, IRS deficiency disrupts the exercise-induced stabilization of PGC-1α at the protein level even if the mRNA levels are unchanged, highlighting the role of IRS in post-transcriptional regulation, such as mTORC1-dependent translation (Riehle et al., 2014). IGF-1 finally enhances fatty acid β-oxidation by upregulating PPARα and carnitine palmitoyltransferase 1B, while simultaneously optimizing glucose metabolism during high-intensity exercise through GLUT4 translocation and hexokinase II activation (Friehs et al., 2001).

### 4.3 Adiponectin (APN)

APN, an adipocytokine inversely correlated with the body mass index, is robustly elevated by high-intensity exercise, particularly in individuals with obesity or metabolic syndrome (Khalafi and Symonds, 2020). APN stimulates mitochondrial biogenesis through the following two synergistic mechanisms: (1) transcriptional activation of PGC-1α by inhibiting AMP-activated protein kinase-dependent histone deacetylase (HDAC), and (2) post-translational deacetylation of PGC-1α by SIRT1, increasing its transcriptional coactivator function (Lin et al., 2013). In murine models, APN administration rescues doxorubicin-induced mitochondrial fragmentation by restoring the fusion-fission balance via mitofusin-2 and dynamin-related protein 1 regulation; conversely, APN knockout mice exhibit defective oxidative phosphorylation and accelerated cardiac aging (Yan et al., 2013). Clinically, exercise-induced APN elevation correlates with improved insulin sensitivity and reduced intramyocardial lipid deposition in patients with diabetes, suggesting that APN is both a biomarker and mediator of exercise benefits in metabolic heart disease (Lee et al., 2011).

The interplay between exercise-induced hormonal regulators (catecholamines, IGF-1, and APN) and PGC-1α orchestrates mitochondrial adaptation and metabolic reprogramming critical for cardiovascular adaptation. β3-AR-mediated eNOS activation amplifies NO/cGMP signaling, driving PGC-1α-dependent mitochondrial biogenesis and respiratory chain optimization (Nisoli et al., 2004). IGF-1, elevated by exercise, coordinates energy substrate utilization via IRS1/2-dependent pathways, stabilizing PGC-1α protein levels through mTORC1-regulated translation and enhancing fatty acid oxidation and glucose metabolism (Riehle et al., 2014; Friehs et al., 2001; Ren et al., 1999). APN, robustly induced by high-intensity exercise, synergistically activates PGC-1α transcriptionally (via AMPK-HDAC inhibition) and post-translationally (via SIRT1-mediated deacetylation), restoring mitochondrial dynamics and improving oxidative phosphorylation (Lin et al., 2013). Collectively, aerobic and resistance training counteract age-related PGC-1α suppression (Neto et al., 2023; Zhang et al., 2024), likely through catecholamine- and APN-driven metabolic reprogramming, establishing a mechanistic framework for precision exercise interventions targeting metabolic and age-related cardiovascular disorders.

## 5 Exercise-mediated attenuation of cardiac fibrosis

**FIGURE 4:**
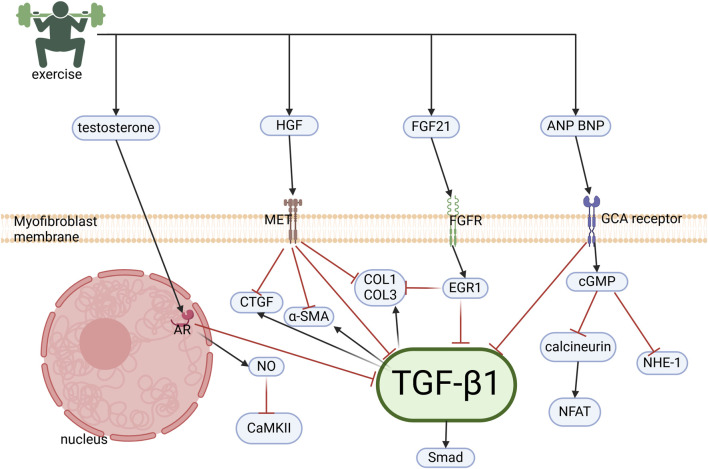
Molecular mechanisms of exercise-induced cardiac anti-fibrotic effects. Exercise induces the cardiac upregulation of testosterone, HGF, FGF21, ANP, and BNP, which collectively suppress fibrogenesis via inhibition of the TGFβ1/Smad pathway in cardiac fibroblasts. Testosterone attenuates collagen synthesis through two mechanisms: (1) Suppression of TGFβ1/Smad signaling and (2) NO-mediated inhibition of Ca^2+^/CaMKII. HGF antagonizes downstream effectors of TGFβ1, including CTGF, preventing ECM deposition. FGF21 upregulates EGR1, which represses collagen and TGF-β1 expression. ANP/BNP signaling through GCA receptors inhibits calcineurin/NFAT, NHE-1, and TGFβ1/Smad cascades, establishing a multi-targeted anti-fibrotic regulatory network.HGF, hepatocyte growth factor; FGF21, fibroblast growth factor 21; ANP, atrial natriuretic peptide; BNP, B-type natriuretic peptide; TGFβ1,Transforming growth factor-β1; NO, nitric oxide; CaMKII, calmodulin-dependent protein kinase II; CTGF, connective tissue growth factor; ECM, extracellular matrix; EGR1, early growth response protein 1; GCA, guanylyl cyclase-A; NHE-1, sodium-hydrogen exchanger 1

Cardiac fibrosis, defined as the pathological replacement of cardiomyocytes with a collagenous matrix following injury or necrosis (Fan and Kassiri, 2021), represents a terminal pathological process in CVD. Notably, exercise has therapeutic effects against fibrosis induced by diverse etiologies, including hypertension, rheumatoid arthritis, and aging (Hong et al., 2022; Peyronnel et al., 2024; Wright et al., 2014). This cardioprotective action is mediated via testosterone, HGF, and fibroblast growth factor 21 (FGF21) signaling pathways (Figure 4).

### 5.1 Testosterone

Transforming growth factor-β1 (TGF-β1), a master regulator of fibrogenesis, drives myofibroblast differentiation, extracellular matrix (ECM) deposition (e.g., collagen I/III and fibronectin), and pro-fibrotic gene activation (e.g., α-smooth muscle actin [α-SMA] and connective tissue growth factor [CTGF]) (Fan and Kassiri, 2021). Exercise-induced testosterone elevation counteracts these processes via a dual mechanism. First, testosterone attenuates TGF-β1-mediated phosphorylation of the Akt/mTOR/4EBP1 axis in cardiac fibroblasts, thereby suppressing proliferation, ECM synthesis, and myofibroblast transdifferentiation (Chung et al., 2014). Second, androgenic signaling increases NO production through eNOS activation. NO exerts anti-fibrotic effects by inhibiting Ca^2+^/calmodulin-dependent protein kinase II, which promotes collagen synthesis via histone deacetylase 4 nuclear translocation (Chung et al., 2021). In fact, preclinical studies reported that rodents with impaired NO synthesis (e.g., eNOS knockout mice) exhibited exacerbated pathological remodeling following exercise, highlighting NO’s critical role in maintaining fibrotic homeostasis (Souza et al., 2007).

### 5.2 HGF

HGF, upregulated by chronic aerobic exercise, exerts anti-fibrotic effects through multiple molecular pathways mediated by its tyrosine kinase receptor c-MET. First, HGF directly suppresses the transcription of TGF-β1 in cardiac fibroblasts, thereby reducing the bioavailability of TGF-β1 and limiting pro-fibrotic signaling (Nakamura et al., 2005). Second, HGF activates the ERK1/2/MAPK signaling cascade, which induces the expression of decorin, a small leucine-rich proteoglycan that binds and sequesters TGF-β1 within the ECM. This spatial neutralization prevents TGF-β1 from engaging its receptor, effectively blunting the downstream Smad2/3 phosphorylation and subsequent fibrotic gene activation (Kobayashi et al., 2003). Finally, HGF attenuates fibrosis by downregulating key markers of myofibroblast activation, including CTGF, a downstream effector of TGF-β1, and α-SMA, a hallmark of fibroblast-to-myofibroblast transition (Gallo et al., 2015). Collectively, these mechanisms underscore HGF’s pivotal role in mitigating ECM remodeling and preserving myocardial compliance under pathological stress.

### 5.3 FGF21

FGF21, a secretory protein, has pleiotropic cardioprotective effects, including preserving myocardial tissue, regulating metabolic homeostasis, suppressing fibrosis, and preventing atrial remodeling (Zhao et al., 2023). Aerobic or endurance exercise significantly elevates circulating FGF21 levels (Bo et al., 2021), which may contribute to the modulation of energy metabolism and ultimately to post-exercise recovery. In young females, serum FGF21 concentrations are significantly elevated following a 2-week exercise regimen (Cuevas-Ramos et al., 2012). Mechanistically, FGF21 activates fibroblast growth factor receptors on cell surfaces to induce early growth response protein 1 expression while suppressing fibrotic mediators, including collagen type I, collagen type III, and TGF-β1 (Li et al., 2021). Furthermore, FGF21 modulates TGF-β1/Smad2/3 and NF-κB signaling pathways, downregulating MMP activity to suppress fibrotic remodeling and scar formation (Ma et al., 2021; Pan et al., 2017). Notably, genetic ablation of FGF21 was shown to abolish the inhibitory effects of aerobic exercise on oxidative stress, ER stress, and apoptosis in myocardial infarction models (Bo et al., 2021).

### 5.4 Natriuretic peptides

Although excessive exercise may induce pressure overload and pathological hypertrophy (Zhou et al., 2020), atrial natriuretic peptide (ANP) and B-type natriuretic peptide (BNP), both established biomarkers of pathological hypertrophy, also play critical roles in blood pressure regulation and fluid-electrolyte homeostasis. Their plasma concentrations increase proportionally to the cardiac output during exercise-induced stress (Yoshiga et al., 2019; Wisén et al., 2011). Importantly, by binding to renal and vascular receptors, these peptides promote natriuresis, diuresis, and vasodilation (Baris Feldman et al., 2023). Within the myocardium, ANP and BNP primarily act through guanylyl cyclase-A receptors to inhibit calcineurin/NFAT, NHE-1, and TGF-β1/Smad signaling pathways (Calvieri et al., 2012), thereby reducing fibroblast proliferation, suppressing inflammatory infiltration, and preventing pathological hypertrophy (Bie, 2018; Kapoun et al., 2004).

The anti-fibrotic effects of exercise are intricately linked to a biphasic dose-response relationship, where the intensity and duration of physical activity play pivotal roles in determining its therapeutic outcomes. At moderate levels, exercise exerts potent anti-fibrotic actions by orchestrating a sophisticated hormonal and growth factor-mediated response (Hong et al., 2022). The delicate balance between exercise’s therapeutic benefits and potential risks becomes apparent at excessive intensities. Overexertion may paradoxically induce pressure overload and pathological hypertrophy, potentially exacerbating ischemia-induced fibrosis (Zhou et al., 2020). This underscores the importance of tailoring exercise regimens to individual patient needs, particularly for those with fibrotic cardiomyopathy. Implementing progressive, low-intensity exercise protocols enables the maximization of therapeutic benefits while minimizing the risk of iatrogenic harm. Such an approach ensures that the anti-fibrotic effects of exercise are harnessed effectively, promoting myocardial compliance and reducing ECM accumulation, without triggering adverse pathological responses (Wright et al., 2014). Ultimately, the judicious prescription of exercise, based on a nuanced understanding of its biphasic dose-response relationship, holds promise as a valuable adjunct therapy in managing cardiac fibrosis.

## 6 Multi-dimensional integration of exercise-induced signaling networks in physiological cardiac hypertrophy

**FIGURE 5:**
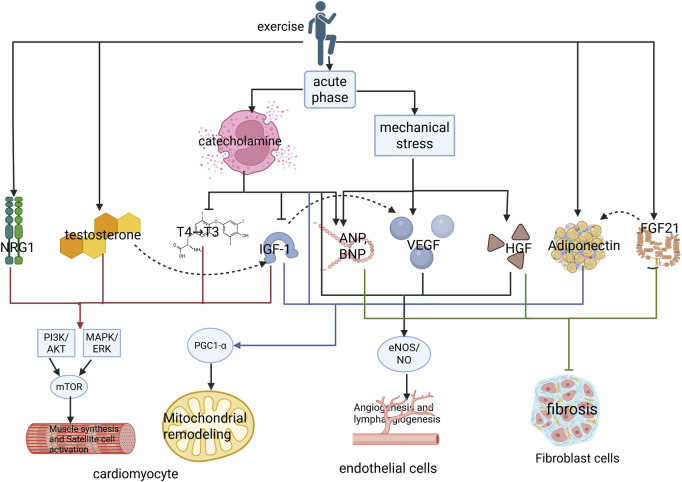
Multidimensional mechanism of exercise induced physiological hypertrophy of the heart. Exercise first promotes the secretion of catecholamines and increases the mechanical stress of the heart through the sympathetic adrenal medullary axis during the acute phase. Catecholamines inhibit the conversion of thyroid hormone T4 to T3 and suppress free IGF-1 by increasing the concentration of IGFBP1. Simultaneously, catecholamines act together with increased mechanical stress to promote the secretion of natriuretic peptides. Catecholamines can also increase mitochondrial energy metabolism and vascular proliferation in the acute phase. During the adaptation period of exercise, the concentration of catecholamines decreases, while concentrations of NRG1, T3, testosterone, IGF-1, VEGF, and FGF21 begin to increase, and FGF21 targets the regulation of adiponectin secretion. IGF-1 may stimulate an increase in VEGF. Finally, NRG1, T3, testosterone, and IGF-1 synergistically activate the PI3K/AKT and MAPK/ERK signaling pathways in cardiomyocytes, which synergistically activate mTOR signaling to promote the synthesis of myocardial fibers and activation of satellite cells. IGF-1 synergistically activates PGC1- α with adiponectin, increasing mitochondrial biogenesis. HGF and VEGF synergistically activate eNOS/NO signaling to promote angiogenesis. HGF, elevated concentrations of testosterone, natriuretic peptide, and FGF21 can inhibit the transformation of fibroblasts into myofibroblasts, protecting the heart from pathological remodeling. IGF-1, insulin-like growth factor-1; PI3K, phosphoinositide 3-kinase; AKT, protein kinase B; ERKs, extracellular signal-regulated kinases; MEK, mitogen-activated protein kinase; NRG1, neuregulin-1; HGF, hepatocyte growth factor; VEGF, vascular endothelial growth factor; eNOS, endothelial nitric oxide synthase; NO, nitric oxide; PGC-1α, peroxisome proliferator-activated receptor gamma coactivator-1α; FGF21, fibroblast growth factor 21.

Exercise impacts the heart across four dimensions: molecular, cellular, systemic, and temporal (Figure 5). During the acute phase of exercise, the sympatho-adrenal axis is activated, triggering catecholamine release. Catecholamines inhibit thyroxine deiodination, thereby reducing the conversion of T4 to T3 during high-intensity exercise (Kobayashi et al., 1966; Nauman et al., 1980). Simultaneously, they stimulate insulin-like growth factor binding protein-1 (IGFBP-1) secretion, consequently decreasing free IGF-1 release and promoting blood glucose elevation (Fernqvist-Forbes et al., 1997). Beyond increased cardiac mechanical stress releasing ANP during exercise, epinephrine can directly induce cardiomyocyte secretion of ANP variants (Sejersen et al., 2022; Huang et al., 1992). Furthermore, catecholamines have been shown to modulate VEGF and IL-6 to enhance angiogenesis (Chakroborty et al., 2009). The early (acute phase) catecholamine (NE) surge activates cardiomyocyte β3-AR, promoting mitochondrial workload, and activates PGC-1α via eNOS/NO signaling, augmenting mitochondrial adaptation during the adaptive phase (Yoshida et al., 2023). These adaptations meet the demands for substrate transport and energy metabolism during exercise. In the adaptive phase following exercise, concentrations of hormones, including T3, IGF-1, NRG1, testosterone, FGF21, and APN increase. IGF-1 has been demonstrated to activate VEGF expression in the heart (Li et al., 2017; Puddu et al., 2021). IGF-1 and NRG1 synergistically regulate cardiac development: IGF-1 is suitable for early expansion of cardiomyocyte numbers, while NRG1 promotes metabolic maturation and electromechanical integration in later stages (Rupe et al., 2017). FGF21 enhances APN production, which subsequently acts on cardiomyocytes to promote mitochondrial bioenergetics. APN partially mediates the protective effects of FGF21 against diastolic dysfunction and cardiac injury induced by HF with reduced ejection fraction in mice (Zhang et al., 2025). VEGF potently drives endothelial cell proliferation and migration, initiating new vessel sprouting and extension. HGF is a potent pro-migratory, pro-morphogenic, and pro-angiogenic maturation factor with significant barrier-stabilizing effects. Both VEGF and HGF respond to stimuli and cooperate to promote angiogenesis (Sulpice et al., 2009; Yang et al., 2015). The interplay among the locomotor, circulatory, and endocrine systems; signal transmission and crosstalk among hormones and growth factors; interactions among cardiomyocytes, endothelial cells, and fibroblasts; and the heart’s responses during both the acute and adaptive phases of exercise collectively drive physiological cardiac adaptation across these dimensions.

Notably, different exercise modalities and intensities may elicit distinct hormonal responses across diverse populations (Table 1). The dose-response relationship between exercise and cardiac health exhibits a “J-shaped curve”: moderate exercise intensity induces physiological cardiac hypertrophy by activating protective molecular pathways, thereby enhancing cardiac function and metabolic adaptability. However, excessive exercise exceeding an individual’s tolerance threshold triggers pressure overload, leading to aberrant elevations in ANP/BNP, activation of the calcineurin pathway, and mitochondrial dysfunction, consequently promoting pathological remodeling and fibrosis (Carraro and Franceschi, 1997; Bernardo et al., 2010). Optimizing intensity and protocols requires the integration of population baseline status, dynamic biomarkers, and individualized progression principles to achieve a precise balance between cardioprotection and risk mitigation. Due to variations among populations and influences from factors. Including sex, genetics, and environment, no study has identified a universal exercise intensity threshold distinguishing physiological from pathological hypertrophy. Nevertheless, based on the data presented in Table 1, we can tentatively estimate that the approximate aerobic exercise intensity risk threshold for healthy adults lies approximately 80%–85% VO_2_max, while the resistance training intensity risk threshold is approximately 85% 1RM. Obese populations may exhibit more pronounced acute responses. Supporting this, zebrafish exercised at 80% of maximal critical swimming speed (Ucrit) for 4 weeks developed pathological cardiac hypertrophy (Zhou et al., 2020). Sixteen weeks of high-intensity endurance training (60 cm/s, 60 min/day) resulted in diastolic dysfunction and increased fibrosis in rats (Benito et al., 2011).

**TABLE 1:** Exercise modality-specific regulation of key hormonal mediators.

Crowd/Model	Motion type	Movement plan	Hormonal response
Healthy young male	Resistance training(RT)	50%1RM,3 h	VEGF↑ (Gustafsson et al., 2005)
	RT	75%–80%1RM	testosterone↑ (Kraemer et al., 1990; Midttun et al., 2024),IGF-1↑ (Schwarz et al., 2016) T3↑, T4↑(acute), T3↑(12 h) (McMurray et al., 1995)
	Endurance training(ET)	<50% VO_2_max	testosterone—— (D'Andrea et al., 2020)
	ET	>60 VO_2_max	IGF-1↑ (Norling et al., 2020), catecholamine↑ (Hansen et al., 2012; Bloom et al., 1976; Bracken et al., 2005) T3↓,T4↑ (Ciloglu et al., 2005; Liewendahl et al., 1992), APN—— (Vu et al., 2007), ANP↑ (Ströhle et al., 2006)
	Acute ET	60% VO_2_max	FGF21↑ (Hansen et al., 2016)
	ET	80% VO_2_max	FGF21↑ (Kim et al., 2013),testosterone↑ (Hayes et al., 2015)
	ET	>80% VO_2_max	T3——,T4—— (Huang et al., 2004), APN—— (Vu et al., 2007), ANP(Acute)↑ (Wisén et al., 2011), ANP(convalescence)↓ (Mandroukas et al., 2011)
	ET (swim)	90% VO_2_max	low-temperature water(<26°C): T4↑ high-temperature water (>26°C): T4↓ (Deligiannis et al., 1993)
	HIIT	90% VO_2_max	T3↑(acute), T3↓(12 h convalescence) (Hackney et al., 2012), HGF↑ (Hamilton et al., 2015)
Healthy young woman	ET	70% VO_2_max	T3——,T4—— (Loucks and Callister, 1993)
	ET	85%VO_2_max	FGF21↑ (Cuevas-Ramos et al., 2012), ANP↑ (Ströhle et al., 2006),BNP↑ (Wisén et al., 2011)
	RT	60%–85%1RM	IGF-1—— (Kraemer et al., 2017; Jiang et al., 2020), catecholamine↑ (Hansen et al., 2012), testosterone—— (Linnamo et al., 2005)
Osteoporotic obese elderly people	RT	60%–70% 1RM, 3 sets × 8–12 repetitions, 10 full-body exercises (8 weeks, 2 times per week)	IGF-1↑↑ (Chen et al., 2017)
	CT	RT (once a week) + AT (once a week, dance aerobics) (8 weeks, 2 times per week)	IGF-1↑ (Chen et al., 2017)
	ET	Moderate-intensity dance (40–45 min per session) (8 weeks, 2 sessions per week)	IGF-1—— (Chen et al., 2017), FGF21↓ (Taniguchi et al., 2016)
Healthy elderly people	ET	Daily walking, combined with moderate-intensity aerobic exercises (12 weeks)	IGF-1↑ (Zouhal et al., 2022), APN↑ (Wang et al., 2015), testosterone—— (Binder et al., 2025)
	ET	ET - Moderate intensity (60%–75% maximum heart rate, 12–52 weeks)	IGF-1↑ (Zouhal et al., 2022)
	ET	60%–120% VO_2_max	VEGF↑ (Vital et al., 2014)
	Acute ET	The second highest intensity (lasting for 20–60 min at a time)	IGF-1↓ (Stein et al., 2021)
	High-intensity interval training(HIIT)	≥85% maximum heart rate (such as 30-s sprint + rest, 5–12 weeks)	IGF-1↑ (Zouhal et al., 2022)
	RT	80%1RM	Testosterone acute↑,adaptive phase—— (Kraemer et al., 2020)
	RT	Moderate to high intensity (70%–85% of 1RM)	IGF-1↑ (Jiang et al., 2020)
	RT + HIIT	Resistance training (75%–80% 1RM) + Cycling sprints (6–9 sets × 60 s, RPE 10)	IGF-1—— (Murray et al., 2025)
Obese adults	Acute RT	70%–85%1RM	catecholamine↓ (Hansen et al., 2012)
	Acute ET	50% VO_2_max	catecholamine ↓ (Hansen et al., 2012)
	ET	60%–80% HRmax	APN↑ (Vu et al., 2007; Khalafi et al., 2023), FGF21—— (Fiorenza et al., 2024)
	RT	60%–85% 1RM	APN↑ (Vu et al., 2007; Khalafi et al., 2023), FGF21↑ (Liu et al., 2024)
	HIIT	85%–90% HRmax	APN↑ (Khalafi et al., 2023), FGF21↓ (Jin et al., 2022) ANP/BNP↑ (Karner-Rezek et al., 2013)
Patients with chronic heart failure (CHF)	ET + RT	50–75% VO_2_max	APN↓ (Van Berendoncks and Conraads, 2011)
	ET	80% VO_2_max	ANP/BNP—— (Larsen et al., 2008)
	ET	100% VO_2_max	ANP/BNP↑ (Bentzen et al., 2004)
Patients with type 2 diabetes mellitus	ET + RT	Moderate strength	APN—— (Zaidi et al., 2021)
	ET	65% VO_2_max	IGF-1↑ (Cheng et al., 2013),APN↑ (Vu et al., 2007),FGF21—— (Kruse et al., 2017),FGF21↓ (Jin et al., 2022), HGF↓ (Xu et al., 2025)
	ET	65%–95% VO_2_max	FGF21—— (Liu et al., 2024), IGF-1↑ (Mazaheri et al., 2025)
	Acute ET	50% VO_2_max	FGF21↑ (Hansen et al., 2016)
	ET	Treadmill: 12 m/min, 5°slope	IGF-1↑ (Li et al., 2022a),APN—— (Vu et al., 2007),ANP↑ (Pan, 2008)
	ET	20 m/min,60 min/day	NRG1↑ (Li et al., 2022b)
	Acute ET	25 m/min	FGF21↑ (Kim et al., 2013)
	RT	Climb stairs,75%1RM	IGF-1↑ (Li et al., 2022a)
	RT	120%Weight load	IGF-1—— (Hatakeyama et al., 2025)
	HIIT	Peak lactate level - 10 mM	VEGF↑ (Morland et al., 2017)
	Whole-body vibration (WBV)	Vertical vibration: 13 Hz frequency, 2 mm amplitude	IGF-1↑ (Li et al., 2022a)
	electrophotoluminescence (ES)	Electrode stimulation: 20 Hz frequency, 1 mA current	IGF-1↑ (Li et al., 2022a)
Mice with myocardial infarction (MI)	ET	10–12 m per min, 60 min per day (4 weeks, 5 days per week)	IGF-1↑ (Feng et al., 2022),VEGF↑, FGF21↑ (Bo et al., 2021; Bo et al., 2023)
	RT	Maximum load: 75%. 3 sets per session, 9 sets per day (4 weeks)	IGF-1↑ (Feng et al., 2022)

↑ : significant increase; ↓ : significant decrease; — no change.

IGF-1, insulin-like growth factor-1; VEGF, vascular endothelial growth factor; APN, adiponectin; ANP, atrial natriuretic peptide; NRG1, neuregulin-1.

Sex and disease-related disparities in exercise-induced hormonal responses significantly influence myocardial adaptation. In males, resistance training (e.g., 75%–80% 1RM) more readily elevates testosterone and IGF-1 levels, promoting cardiomyocyte hypertrophy and suppressing myocardial apoptosis by activating the PI3K/Akt pathway. Conversely, females exhibit greater sensitivity to endurance training-induced increases in FGF21. Under disease states, obesity/diabetes attenuates catecholamine and FGF21 responsiveness; however, endurance training can still improve myocardial mitochondrial function via the IGF-1 pathway. In patients with heart failure (HF) patients, post-exercise elevations in ANP/BNP following high-intensity exercise may exacerbate cardiac loading. Conversely, post-myocardial infarction exercise promotes angiogenesis via IGF-1/VEGF signaling. Notably, individuals with sarcopenic obesity require higher-intensity resistance training to elevate IGF-1 levels.

## 7 Conclusion and prospect

This study reviews the molecular mechanisms by which exercise induces physiological cardiac hypertrophy, underscoring the central role of the multi-dimensional regulation of hormonal and growth factor networks in cardiovascular protection. Future studies should develop dynamic biomarker panels to personalize exercise dosing in HF, leveraging the biphasic responses of exercise-induced mediators revealed in this review. A stratified framework monitoring safety thresholds (e.g., ANP/BNP and troponin for pressure overload and injury, respectively) and efficacy signals (e.g., VEGF-C/HGF for angiogenesis; FGF21/APN for mitochondrial adaptation, lipid metabolism, and insulin sensitivity; and TGF-β1 suppression for anti-fibrosis) can guide intensity titration. Integrating wearable hemodynamic sensors with serial biomarker profiling (pre-/post-exercise) could enable adaptive algorithms—such as moderate continuous training for patients with HF with reduced ejection fraction who exhibit IGF-1 resistance versus carefully dosed high-intensity interval training for those with obesity who have HF with preserved ejection fraction when APN/FGF21 ratios indicate metabolic responsiveness—thereby balancing cardioprotection while minimizing pathological strain. Multicenter trials validating these panels are essential to translate mechanistic insights into precision exercise prescriptions for HF subpopulations.

Furthermore, circulating biomarker levels may predict athletic ability in patients with HF. As demonstrated in preclinical models, baseline NRG1 deficiency correlates with impaired cardiac repair capacity, while exercise-induced NRG1 elevation (>40% from baseline) enhances ErbB4-mediated cardiomyocyte proliferation and metabolic maturation—key mechanisms for functional recovery. Clinical validation should determine whether pre-intervention NRG1 thresholds can identify patients most likely to benefit from moderate-intensity endurance protocols, particularly those with ischemic cardiomyopathy, where NRG1/ErbB signaling is essential for angiogenesis and fibrosis regression.

The genetic polymorphisms of some important receptors cannot be ignored due to their impact on exercise outcomes and cardiovascular responses. The IGF1R rs1464430 polymorphism exhibits associations with exercise type: the AA genotype appears more favorable for endurance-oriented sports, while the C allele is a distinguishing feature among strength/power athletes (Ben-Zaken et al., 2015). The β_2_-AR Gln27Glu polymorphism significantly influences the therapeutic response to carvedilol in patients with chronic HF, with Glu27 homozygotes exhibiting significantly greater improvements in systolic/diastolic function and exercise hemodynamics (Metra et al., 2010). β_2_-AR Arg16Gly homozygosity is associated with enhanced muscle mass and strength gains in athletes (Jenkins et al., 2018). Furthermore, the VEGFR2 His472Gln polymorphism enhances aerobic endurance by increasing VO_2_max and the proportion of slow-twitch fibers (Ahmetov et al., 2009).

Beyond the mediators discussed above, emerging research highlights novel regulators involved in exercise-induced cardiac adaptation. Nuclear factor erythroid 2-related factor 2 protects the heart against oxidative stress during exercise, attenuating pressure overload-induced pathological cardiac hypertrophy and dysfunction (Ni et al., 2025). MicroRNA-223-3p and Myostatin have been identified as novel biomarkers indicative of acute exercise and training-induced cardiac adaptation (Fernandez-Vivero et al., 2025; Heineke et al., 2010; Lenk et al., 2012). Irisin mediates multiple cardioprotective effects of exercise, including cardiac angiogenesis, anti-inflammation, energy metabolism optimization, and mitophagy (Guo et al., 2024). Furthermore, IL-6 plays a functional role in mediating exercise-induced improvements in cardiac contractile function (Jønck et al., 2024). These newly identified mediators warrant focused investigation in future research to refine our understanding of the molecular mechanisms underpinning cardiac adaptation to exercise.

## Abbreviations

CVDs, cardiovascular diseases; IGF-1, insulin-like growth factor-1; IGF1R, insulin-like growth factor-1 receptor; IRS1, insulin receptor substrates 1; PI3K, phosphoinositide 3-kinase; Akt, protein kinase B; ERKs, extracellular signal-regulated kinases; MEK, mitogen-activated protein kinase; NRG1, neuregulin-1; HGF, hepatocyte growth factor; VEGF, vascular endothelial growth factor; PlGF, placental growth factor; eNOS, endothelial nitric oxide synthase; NO, nitric oxide; LEC, lymphatic endothelial cell; MMPs, matrix metalloproteinases; β3-AR, β3-adrenergic receptor; PGC-1α, peroxisome proliferator-activated receptor gamma coactivator-1α; PPARs, peroxisome proliferator-activated receptor α; ERRs, estrogen-related receptor; APN, adiponectin; HDAC, histone deacetylase; FGF21, fibroblast growth factor 21; TGF-β1, transforming growth factor-β1; ECM, extracellular matrix; α-SMA, α-smooth muscle actin; CTGF, connective tissue growth factor; ANP, atrial natriuretic peptide; BNP, B-type natriuretic peptide; HF, heart failure.
